# Improving the criticism of antiscientific medicine: a commentary on Steven K Baker “Medical Lysenkoism”

**DOI:** 10.1111/jep.14191

**Published:** 2024-10-21

**Authors:** Piersante Sestini

**Affiliations:** ^1^ Department of Medical and Surgical Sciences and Neurosciences University of Siena Siena Italy

**Keywords:** epistemology, evidence‐based medicine, medical ethics, philosophy of medicine, science

The essay on medical Lysenkoism recently published by the journal^1^ is engaging, well‐written, original, dense, thought‐provoking, and even entertaining. While it presents original ideas and is dense with insightful analysis, its persuasiveness appears to be weakened by an overall insufficiency of internal consistency and logical coherence.

For readers unfamiliar with the Lysenko affair, it was a troubling chapter in the history of the Soviet Union and its satellite parties from the 1930s through the 1960s. Trofim Denisovič Lysenko, an Ukrainan agronomist with little academic background, with the backing of Joseph Stalin and the Communist Party, imposed his own unscientific “agrobiology” not only in the government policies, but even in the academy, refusing any independent evaluation of its claims, banning genetic research studies as “bourgeois science”, and leading to disgrace, persecution, exile, inprisonment and even death of many accomplished genetists.^2^

The author begins with a clear premise: medicine has as its primary purpose the minimization of suffering associated with both psychological and physical disease. It is composed of both science and art (though the author likely means “human judgment” or “human faculty,” as most arts are no less technical than medical practice, yet the term “art” is commonly used in this context). If medicine, like any scientific practice, fails to align itself with reality (what the author calls “truth”), it cannot function effectively. The author introduces the notion of “medical Lysenkoism,” where the politicization of medicine replaces the open‐ended goal of inquiry‐based knowledge with the pre‐determined goal of ideology‐based political ends. The comparison with the tragic history of geneticists under Lysenko is compelling, original, and effective. Furthermore, it resonates with a longstanding debate on the dignity of medicine and its independence from unproven ideologies, a debate traceable to the earliest documents of Greek medicine, such as the 5th‐century BCE text “Ancient Medicine,” possibly of Pythagorean origin.^3^ Medicine is not justified by aprioristic theories, but by the results of their application.

However, the essay takes a sudden turn, where the necessity for the physician/scientist to adhere to a belief system that accepts the existence of reality and its susceptibility to human understanding (albeit with limitations) becomes conflated with the necessity that reality itself must be knowable and predictable—an expectation that clearly exceeds human capabilities. Additionally, the author somewhat dogmatically excludes from knowable reality anything that is not unchanging (not “fictile”) or is subjective. This stance is problematic because, as the author admits, the world itself is in constant flux: continents shift, environments evolve, diseases transform, and human tools and knowledge change. Scientific knowledge, therefore, should not be seen in Whig historical terms as an unstoppable march toward the full discovery of Truth—an idea uncomfortably similar to Stalin's and Lysenko's path to “the sun of the future”—but rather as a continuous adaptation of our understanding to current phenomena using the best available evidence at any given time. As Claude Bernard once stated, “*Quand nous faisons une théorie générale dans nos sciences, la seule chose dont nous soyons certains, c'est que toutes ces théories sont fausses absolument parlant”*.* ^,^ ^4^

Another dogmatic and disruptive statement is that for medicine to be scientific, it must minimize “the subjective as it is unmeasurable even if it is statistically analyzable.” This contradicts the initial claim that medicine aims to treat both illness and disease, and it excludes from medical consideration phenomena such as pain or breathlessness, and most psychiatric disorders. It also conflicts with later appeal to the human medical relationship, evidence‐based medicine, and personalized medicine, which all involve the understanding and interpretation of patient narratives, feelings, beliefs, preferences, and values.^5^, ^6^, ^7^

From this point on, the essay's discussion becomes less clearly related to its declared theme. One might expect examples of “Lysenkoist medicine” pursuing utopian goals while disregarding falsification by the analysis of its results. However, in the first example the author discusses faculty recruitment issues in academia, arguing about the incompatibility of the introduction (apparently for ideological reasons), of proportional criteria with “pure meritocracy.” The argument is lengthy and somewhat pedantic, including some unnecessary mathematical formulas. It seems obvious that any “pure” criterion loses its purity when mixed with others. What is missing, however, is evidence supporting the claimed loss of effectiveness due to this “impurity,” especially given that the limitations of a “pure meritocracy” were exposed at the very time when this neologism was first coined.^8^ While the critique of methods proposed to promote equity, but which may have opposite effects, seems reasonable, the arguments could be stronger if they were more closely tied to the actual problem at hand (apparently, the reduced access of minorities to academic culture) and possible solutions (such as improving opportunities for these groups), with a comparison of outcomes. As presented, the appeal to “pure meritocracy” itself seems ideological, likely against the author's intentions.

The essay continues in this vein, confronting a variety of ideologies and with activism in general, exaggerating differences to create unsolvable dichotomies and paradoxes rather than discussing their aims and results, in an attempt to solve problems with anything other than the complete defeat of the opposing side. Collateral damage in this process include public health, epidemic control, sex reassignment surgery (and possibly surgery as a whole, since many other surgical procedures also have irreversible effects and are obviously affected by uncertainty), the ethical principles of patient autonomy and justice, qualitative research and reflexivity.

The more convincing argument against the proposed ideologies is summarized in the sentence “There is, thus, a futile storm pressing toward an elusive goal mired in the miasma of ill‐description and recession”, which can be ascribed to all the covered topics, as presented. Nevertheless while the scientific background of “agrobiology” was at least dubious† (although it still has some supporters for some of its aspects^9^), the main scientific issues were not so much its purported aims or the theory itself, but the refusal to accept its results (inflating and even faking them), and any independent evaluation.^10^ An even more severe problem was its essential obscurantism: Lysenkoists, using political arguments, made it impossible to pursue scientific knowledge with alternative scientific approaches, damaging not only soviet agriculture, but the development of genetics and biology for decades.^11^, ^12^ Yet, while examples of these antiscientific practices could be still found today, the essay does not give them due consideration.

Ultimately, “medical Lysenkoism”, as designed, may feel more like a straw man used for ideological debate than a useful interpretive category.

## CONFLICT OF INTEREST STATEMENT

The author declares no conflicts of interest.

## Data Availability

Not applicable.

## Footnotes

* “When we formulate a general theory in our sciences, the only thing we can be certain of is that all these theories are false, in absolute terms”

† For Thomas Kuhn, Lysenko's blunder involved believing in an immature theory, one that wasn't established within an existing paradigm. For Karl Popper, the problem was Lysenko's inability to acknowledge the errors that were happening all around him, and accept that his theory had been falsified.^9^
